# Comparison of Rigid and Soft-Brace Treatments for Acute Osteoporotic Vertebral Compression Fracture: A Prospective, Randomized, Multicenter Study

**DOI:** 10.3390/jcm8020198

**Published:** 2019-02-06

**Authors:** Tsuyoshi Kato, Hiroyuki Inose, Shoichi Ichimura, Yasuaki Tokuhashi, Hiroaki Nakamura, Masatoshi Hoshino, Daisuke Togawa, Toru Hirano, Hirotaka Haro, Tetsuro Ohba, Takashi Tsuji, Kimiaki Sato, Yutaka Sasao, Masahiko Takahata, Koji Otani, Suketaka Momoshima, Ukihide Tateishi, Makoto Tomita, Ryuichi Takemasa, Masato Yuasa, Takashi Hirai, Toshitaka Yoshii, Atsushi Okawa

**Affiliations:** 1 Department of Orthopaedics, Graduate School, Tokyo Medical and Dental University, Tokyo 108-0075, Japan; kato.orth@tmd.ac.jp (T.K.); yuasa.orth@tmd.ac.jp (M.Y.); hirai.orth@tmd.ac.jp (T.H.); yoshii.orth@tmd.ac.jp (T.Y.); okawa.orth@tmd.ac.jp (A.O.); 2 Department of Orthopaedics, Ome Municipal General Hospital, Tokyo 198-0042, Japan; 3 Department of Orthopaedics, Kyorin University, Tokyo 181-8611, Japan; ichimura@ks.kyorin-u.ac.jp; 4 Department of Orthopaedic Surgery, Nihon University, Tokyo 173-8610, Japan; tokuhashi.yasuaki@nihon-u.ac.jp; 5 Department of Orthopedic Surgery, Graduate School of Medicine, Osaka City University, Osaka 545-8585, Japan; hnakamura@med.osaka-cu.ac.jp (H.N.), hoshino717@gmail.com (M.H.); 6 Department of Orthopaedic Surgery, Hamamatsu University of Medicine, Shizuoka 431-3192, Japan; daisuketogawa@wish.ocn.ne.jp; 7 Department of Orthopedic Surgery, Niigata University Medical and Dental General Hospital, Niigata 951-8520, Japan; thirano@med.niigata-u.ac.jp; 8 Department of Orthopaedic Surgery, University of Yamanashi, Yamanashi 409-3898, Japan; haro@yamanashi.ac.jp (H.H.); tooba@yamanashi.ac.jp (T.O.); 9 Department of Orthopaedic Surgery, Kitasato University Kitasato Institute Hospital, Tokyo 108-8642, Japan; tsuji9@gmail.com; 10 Department of Orthopaedic Surgery, Kurume University School of Medicine, Kurume University, Fukuoka 830-0011, Japan; kimiaki@med.kurume-u.ac.jp; 11 Department of Orthopaedic Surgery, Graduate School, School of Medicine, St. Marianna University, Kanagawa 216-8511, Japan; sasaospine@marianna-u.ac.jp; 12 Department of Orthopaedic Surgery, Hokkaido University Graduate School of Medicine, Hokkaido 060-8638, Japan; takahatamasahiko@hotmail.co.jp; 13 Department of Orthopaedic Surgery, Fukushima Medical University School of Medicine, Fukushima 960-1295, Japan; kojiotani1964@gmail.com; 14 Department of Diagnostic Radiology, Center for Preventive Medicine, Keio University School of Medicine, Tokyo 160-8582, Japan; momo@rad.med.keio.ac.jp; 15 Department of Diagnostic Radiology, Medical Hospital, Tokyo Medical and Dental University, Tokyo 113-8510, Japan; ttisdrnm@tmd.ac.jp; 16 Clinical Research Center, Tokyo Medical and Dental University, Tokyo 113-8510, Japan; tomita.crc@tmd.ac.jp; 17 Department of Orthopaedic Surgery, Kochi Medical School, Kochi 783-8505, Japan; takemasa-koc@umin.ac.jp

**Keywords:** osteoporosis, osteoporotic vertebral compression fracture, brace, quality of life, spinal deformity

## Abstract

While bracing is the standard conservative treatment for acute osteoporotic compression fracture, the efficacy of different brace treatments has not been extensively studied. We aimed to clarify and compare the preventive effect of the different brace treatments on the deformity of the vertebral body and other clinical results in this patient cohort. This multicenter nationwide prospective randomized study included female patients aged 65–85 years with acute one-level osteoporotic compression fractures. We assigned patients within four weeks of injury to either a rigid-brace treatment or a soft-brace treatment. The main outcome measure was the anterior vertebral body compression percentage at 48 weeks. Secondary outcome measures included scores on the European Quality of Life-5 Dimensions (EQ-5D), visual analog scale (VAS) for lower back pain, and the Japanese Orthopaedic Association Back Pain Evaluation Questionnaire (JOABPEQ). A total of 141 patients were assigned to the rigid-brace group, whereas 143 patients were assigned to the soft-brace group. There were no statistically significant differences in the primary outcome and secondary outcome measures between groups. In conclusion, among patients with fresh vertebral compression fractures, the 12-week rigid-brace treatment did not result in a statistically greater prevention of spinal deformity, better quality of life, or lesser back pain than soft-brace.

## 1. Introduction

Owing to the aging population, the prevalence of osteoporosis has increased rapidly, and the number of patients with fragile fractures has also increased. Among the fragile fractures, vertebral fractures are the most common [1]. Indeed, the Rotterdam study showed an incidence of 19.6/1000 person-years at ages over 75 years for women [2].

In the acute stage, osteoporotic vertebral fractures cause severe back pain, disability in activities of daily living (ADL), and deterioration of the quality of life (QOL), but their life prognosis has been considered relatively good. However, changes in anterior vertebral height caused by osteoporotic vertebral compression fractures can progress with time and result in spinal deformities, such as degenerative kyphosis [3]. Hyper kyphotic alignment itself imposes stress on the anterior spinal column, increases the risk of new compression fractures, and also disrupts normal body balance, resulting in an increased risk of falls and other fractures [3]. Moreover, other factors that can arise from spinal deformity after osteoporotic vertebral fractures, such as reduced lung function, loss of independence, depression, and chronic pain can negatively impact life [3,4,5]. Thus, when treating osteoporotic vertebral compression fracture patients, reducing the degree of spinal deformity as much as possible is important for improved QOL after fracture.

The current standard treatment of acute vertebral compression fractures includes continuous bracing for up to 6 to 12 weeks until the acute pain resolves [4,6]. The purposes of braces are controlling back pain by limiting motion, stabilizing injured structures by immobilizing the spine, increasing trunk muscle strength, improving respiratory function, and providing pressure to provide correction or prevent progression of a deformity [7,8,9]. Rigid braces are commonly custom-made to provide the most support to the spine. Accordingly, the material cost of a rigid brace is more expensive than a soft brace or ready-made brace. However, complications regarding rigid-brace treatments such as pressure injury, discomfort, and emotional distress have been reported [10,11,12]. The problem is that the current evidence remains insufficient in terms of the efficacy of brace treatment for acute vertebral compression fractures because of the few prospective randomized studies [8,9,10,13,14,15,16]. Besides, even the results of prospective, randomized studies are inconsistent; for example, one study demonstrated that the rigid-brace treatment prevented further vertebral deformity compared to non-brace treatment [13], whereas another study demonstrated that the Oswestry Disability Index scores and progression of spinal deformity were similar among non-brace, soft-brace, and rigid-brace groups [14]. Furthermore, one study demonstrated that patients treated with dynamic orthosis had a greater reduction in pain compared to three point orthosis [9], whereas another study demonstrated that the pain level and functional mobility level were similar between dynamic orthosis and soft lumbar orthosis [15]. As a result, the duration and selection of the type of brace, such as rigid brace, soft brace, or ready-made brace, were at the discretion of each physician [16].

Based on the results of a previous pilot study [13], we hypothesized that a rigid brace is superior to a soft brace in preventing further spinal deformity. This large randomized controlled trial aimed to clarify and to compare the preventive effect of the different brace treatments on the deformity of the vertebral body and other clinical results in patients with vertebral compression fractures.

## 2. Experimental Section

### 2.1. Trial Design

We conducted a nationwide, multicenter, open-label, clinical superiority trial in which patients with acute osteoporotic compression fractures were randomly assigned, in a 1:1 ratio, to wear either a rigid brace or soft brace for 12 weeks. This trial was approved by the Tokyo Medical and Dental University ethics review boards (1862), and all participants provided oral and written informed consent. The trial took place in 71 Japanese hospitals. We enrolled patients aged between 65 and 85 years who had received a diagnosis of one fresh osteoporotic compression fracture between T10 and L2 within four weeks of injury. The diagnosis of compression fracture was based on the presence of acute lower back pain and the findings present in their plain lateral radiograph or magnetic resonance imaging (MRI). Exclusion criteria included more than two previous compression fractures from T10 to L2, previous spinal surgery, neurological deficits, inability to complete questionnaires, and serious comorbid conditions such as serious cancer and infection.

Simple randomization was performed with the use of a secure, encrypted, web-based system that enabled computer-generated random treatment assignment. Randomization was stratified according to the age.

### 2.2. Interventions

**Figure 1:**
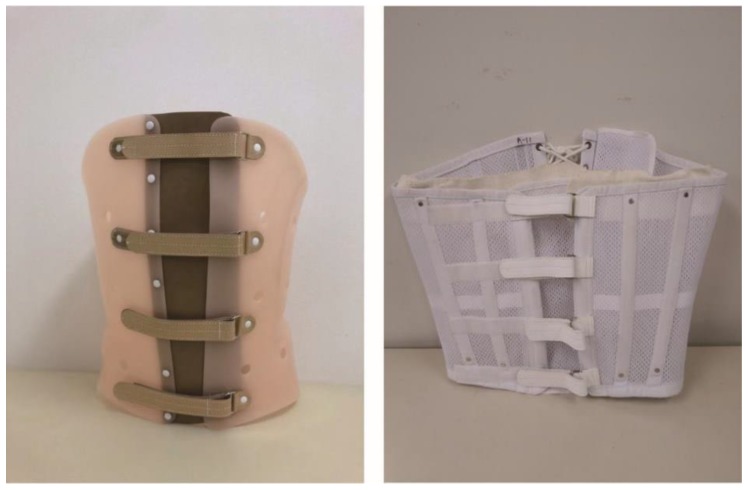
Braces. Rigid brace (**left**) and soft brace (**right**): anterior view.

Physicians explained the pathological condition to each patient, and upon patient approval, each patient was invited to participate in this study. Patients wore ready-made braces until a custom-made thoracolumbar sacral rigid or soft brace was applied. Patients in the rigid-brace group received a rigid thoracolumbosacral orthosis, which is a single-piece molded plastic brace with an opening on the front (Figure 1). Patients in the soft-brace group received a soft thoracolumbosacral orthosis, which is made from an elastic cotton/nylon material and has steel stays with an opening on the front (Figure 1). In both the rigid-brace and soft-brace groups, braces were to be worn at all times. All participants were instructed to wear the brace for a total of 12 weeks. The brace compliance and use of analgesics were self-reported by the patients during the follow-up assessments. With regard to the use of anti-osteoporosis treatment, patients were only allowed the same medication used before injury or newly prescribed active vitamin D. The participants did not receive financial support for the treatments, including brace application and pain medication.

### 2.3. Data Collection and Outcomes

Outcomes of this trial were evaluated using radiographic findings and patient-reported data obtained from validated questionnaires. The primary outcome was the anterior vertebral body compression percentage (AVBCP) at 48 weeks [17], which was defined as the ratio between the vertical height of the compressed anterior section of the injured vertebral body and the posterior vertebral body height at the same level. AVBCP was measured independently at 0, 12, 24, and 48 weeks after the application of the brace, by two of the co-authors, who were unaware of the treatment method. The mean value of the two evaluators was used. Secondary outcomes were scores on the European Quality of Life-5 Dimensions, 3-Level questionnaire (EQ-5D-3L; which ranges from −0.111 to 1, with higher scores indicating better QOL) [18], visual analog scale (VAS) for lower back pain (which ranges from 0 to 100, with higher scores indicating severe pain) [19], and the Japanese Orthopaedic Association Back Pain Evaluation Questionnaire (JOABPEQ; which scores pain-related disorders, lumbar spine function, walking ability, social life function, and mental health; each score ranging from 0 to 100, with higher scores indicating better function) [20]. JOABPEQ is based on the Roland–Morris Disability Questionnaire and Short Form 36. These questionnaires were obtained at a regular hospital visit (0, 12, and 48 weeks after the application of the brace), but completed without the assistance of the surgeon or any other person involved in the trial. In addition, the patients responded to questions related to brace compliance and use of analgesics at 4, 8, and 12 weeks and at 4, 8, 12, 24, and 48 weeks, respectively. Lateral radiography was performed at 0, 12, 24, and 48 weeks after the brace application. All data were collected by a blinded clinical research assistant.

### 2.4. Statistical Analysis

The outcome analysis was performed by comparisons between the rigid-brace and soft-brace groups. On the basis of the results of the previous study of radiological AVBCP [13], we performed a power analysis. A total of 110 patients were needed to detect a significant difference in the radiological AVBCP as a primary outcome, with >80% statistical power. Allowing 20% loss during follow-up, we decided to enroll >140 patients into each arm, yielding a minimum of 280 patients in total.

Differences between the two treatment groups were analyzed using the Mann–Whitney U tests for continuous variables and the Fisher’s exact tests for nominal variables. As an ancillary analysis, we assessed the difference of AVBCP between each follow-up period and the correlation between AVBCP and secondary outcome measures in all patients at 48 weeks. All statistical analyses were performed with EZR (Saitama Medical Center, Jichi Medical University, Saitama, Japan), which is a graphical user interface for R (The R Foundation for Statistical Computing, Vienna, Austria) [21,22]. We imputed the missing data measurements with data obtained by the Multiple Imputation by Chained Equations package in R. We chose 20 iterations for the multiple imputation. All tests were 2-sided, and *p*-values < 0.05 were considered significant.

### 2.5. Availability of Data and Material

The datasets generated and/or analyzed during the current study are not publicly available due to conditions of ethical approval but are available from the corresponding author on reasonable request.

### 2.6. Trial Registration

UMIN000014876.

## 3. Results

### 3.1. Baseline Characteristics of the Patients

**Figure 2:**
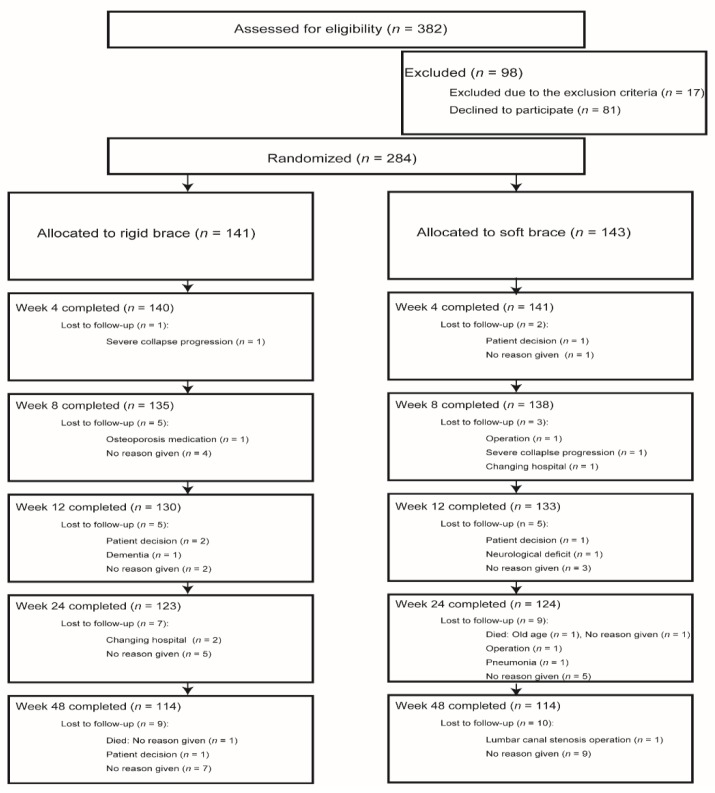
Participant flow through the study. Consolidated Standards of Reporting Trials flow diagram showing patient disposition.

From August 2014 to September 2016, a total of 382 patients were assessed for eligibility for the study. A total of 284 patients met the inclusion criteria and participated in this study, and were randomly assigned to a study group. A total of 141 patients were assigned to the rigid-brace group, whereas 143 patients were assigned to the soft-brace group (Figure 2). The baseline characteristics of the patients are shown in Table 1. There are no significant differences between the two treatment groups in any of the pretreatment variables.

The rate of missing data was similar among the groups at all follow-up time points, and there was no crossover among the groups at any follow-up time points.

### 3.2. Primary Outcome

**Figure 3:**
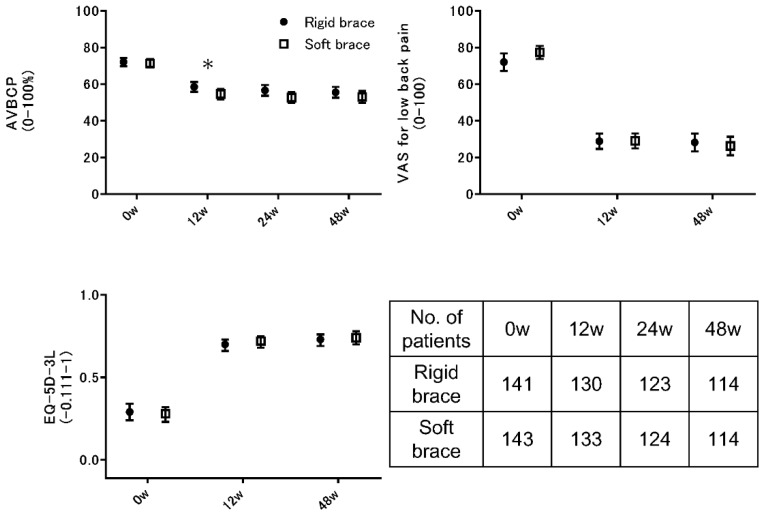
Temporal trends in the main study outcomes. Anterior vertebral body compression percentage (0 to 100, with higher scores indicating less deformity), visual analog scale (VAS) for lower back pain (0–100, with higher scores indicating severe pain), and European Quality of Life-5 Dimensions 3-Level questionnaire (EQ-5D-3L, −0.111 to 1, with higher scores indicating better quality of life). Shown are the means with 95% CIs at baseline and each follow-up. * *p* < 0.05; AVBCP, anterior vertebral body compression percentage; VAS, visual analog scale; EQ-5D-3L, European Quality of Life-5 Dimensions 3-Level questionnaire; CI, confidence interval.

There was no significant difference in the AVBCP at 48 weeks between the patients with rigid-brace treatment and those with soft-brace treatment (Figure 3). The mean AVBCP in the rigid-brace group was 55.5 vs 53.0 in the soft-brace group, resulting in a difference of −2.5 (95% confidence interval (CI), −7.0 to 1.8) in favor of rigid-brace treatments (*p* = 0.20). Although there was also no significant difference in AVBCP at 24 weeks (*p* = 0.07), patients in the rigid-brace group had a significantly higher AVBCP than those in the soft-brace group at 12 weeks (*p* = 0.04) (Figure 3 and Table 2). There was no significant difference in the rate of aggravation of the AVBCP at 48 weeks (91.2% in the rigid-brace group vs 91.2% in the soft-brace group; *p* > 0.05). 

The primary outcome data were complete in 80% of the starting cases (228 of 284 available study participants provided final outcome data).

The difference calculated as the mean of the soft-brace group minus the mean of the rigid-brace group; for AVBCP, a negative value denotes a more favorable outcome with rigid-brace treatment, and a lower score indicates greater spinal kyphosis.

### 3.3. Secondary Outcomes

All scores (VAS, EQ-5D-3L, and JOABPEQ) significantly improved after the application of either brace (Figure 3 and Table 2). Secondary outcome measures showed no significant differences between groups in the results of the VAS score for lower back pain, EQ-5D-3L, and JOABPEQ. The mean VAS score for lower back pain at 48 weeks in the rigid-brace group was 28.2 vs 26.3 in the soft-brace group, resulting in a difference of −1.9 (95% CI, −8.7 to 5.1) in favor of soft-brace treatments (*p* = 0.43). The mean EQ-5D-3L score at 48 weeks in the rigid-brace group was 0.73 vs 0.74 in the soft-brace group, resulting in a difference of 0.01 (95% CI, −0.04 to 0.07) in favor of soft-brace treatments (*p* = 0.67). The mean JOABPEQ pain-related disorder score at 48 weeks in the rigid-brace group was 57.3 vs 60.6 in the soft-brace group, resulting in a difference of 3.3 (95% CI, −4.4 to 11.0) in favor of soft-brace treatments (*p* = 0.43). 

### 3.4. Ancillary Analysis

A post hoc analysis of the total patients showed a significant difference in AVBCP between 0 week and 12 weeks (*p* < 2 × 10^−16^), 0 week and 24 weeks (*p* < 2 × 10^−16^), 0 week and 48 weeks (*p* < 2 × 10^−16^), 12 weeks and 24 weeks (*p* = 2.7 × 10^−5^), and 12 weeks and 48 weeks (*p* = 0.0001). However, there was no significant difference in AVBCP between 24 and 48 weeks (*p* = 0.85). Interestingly, a post hoc analysis of the total patients showed that there was a significant inverse correlation between AVBCP and VAS for lower back pain (*p* = 0.003) and a significant correlation between AVBCP and pain-related disorders of JOABPEQ (*p* = 0.0002) at 48 weeks (Table 3). However, we did not observe any correlation between AVBCP and other factors (Table 3).

### 3.5. Brace Compliance and the Use of Analgesics

In terms of brace compliance, there were no significant differences between the rigid-brace and soft-brace groups at 4 (*p* = 0.30), 8 (*p* = 0.49), and 12 weeks (*p* = 0.77) (Table 4). Use of analgesics decreased in both groups at all time points, with no statistically significant differences between the groups (Table 5).

### 3.6. Complications

Seven and nine patients in the rigid-brace and soft-brace groups, respectively, developed new vertebral fractures, showing no significant difference between groups (odds ratio 0.78 (95% CI 0.23 to 2.42) and a percentage difference in rates of −1.3% (95% CI, −6.7% to 4%) in favor of the rigid brace). During the follow-up period, none of the patients in the rigid-brace group underwent a secondary operation, whereas two patients in the soft-brace group did; however, this was not significantly different between the groups (odds ratio 0 (95% CI 0 to 5.39) and a percentage difference in rates of −1.4% (95% CI, −3.3% to 0.5%) in favor of rigid brace).

## 4. Discussion

This prospective, randomized controlled trial demonstrates that the radiological outcomes of patients treated with a rigid brace or a soft brace for acute vertebral compression fractures are not significantly different at 48 weeks after the application of the brace, although the rigid-brace treatment resulted in better prevention of spinal deformity than the soft-brace treatment at 12 weeks. No differences in the secondary outcomes, including improvement of back pain, QOL status (EQ-5D-3L), and JOABPEQ, were found between the groups at the 48-week follow-up.

It is generally agreed that lumbar compression fractures without neurological deficits should be treated with braces, although there is no consensus on the brace duration [16]. A multicenter cohort study reported that 19.6% of patients did not achieve fracture union at six months [23]. Another prospective study showed that 13.6% of the fresh compression fractures resulted in a delayed union at six months of follow-up [24]. If the external fixation were to be removed, AVBCP would deteriorate especially in delayed union patients. Indeed, AVBCP significantly decreased from 12 weeks to 24 weeks in this study, suggesting that a uniform brace period of 12 weeks may not be sufficient. On the basis of our results, to prevent further spinal deformity, we recommend that the brace treatment period should be determined individually until bony union is confirmed, not <12 weeks. However, an adequate brace period to prevent further spinal deformity is still not identified, and future investigations regarding this are warranted.

Regarding clinical outcomes, no differences were found in the improvement of back pain, QOL status (EQ-5D-3L), and JOABPEQ between the groups at all time points. Moreover, regarding pain related to compression fractures, there were no significant differences between groups in the use of analgesics throughout the study. Collectively, both braces were equally effective for managing pain and disability of QOL from compression fractures.

Health-related QOL has been affected by spinopelvic malalignment [25]. However, we did not find an association between QOL (EQ-5D-3L) and AVBCP in this study. These results may indicate that the existence of one vertebral fracture does not always deteriorate a patient’s QOL even if the local spinal deformity is severe. However, given that spinal kyphosis is associated with an increased risk of fall and new fractures, it is likely that a patient’s condition may worsen in the future. Thus, without a longer follow-up duration, it may be difficult to gauge the impact of residual spinal deformity after compression fracture on the QOL in this short-term follow-up study of 48 weeks. 

We identified a significant inverse association between AVBCP and VAS for low back pain and a significant association between AVBCP and JOABPEQ category pain-related disorders (Table 3). These findings were consistent with the previous notion that patients with low lumbar lordosis presented with back pain [26]. Thus, to reduce the chronic lower back pain after a compression fracture, doctors should develop the appropriate treatment strategy to prevent further spinal deformity.

No significant differences were observed in brace compliance between groups. Although compliance with rigid-brace treatment has been reported to be poor [27], our results showed that patients are more compliant with wearing a rigid brace if doctors provide proper instructions. Regarding the number of patients requiring secondary surgery, there were no significant differences between groups (none in the rigid-brace group, two in the soft-brace group), and the number is quite small. Considering that the VAS score and QOL were improved with time, we do not recommend that patients with acute vertebral fractures undergo vertebroplasty early. In our opinion, vertebroplasty for acute vertebral fractures should be indicated only in a selected subgroup of patients with insufficient pain relief due to a delayed union after the initial conservative treatment.

This study has several limitations. First, physicians and patients could not be blinded to the treatment after they were assigned to a specific study group, but radiographic evaluators at all outcome points were blinded. Second, there has been no consensus on the minimum clinically significant difference of spinal deformity. Therefore, a further study confirming the effect of spinal deformity on back pain and QOL is needed. Third, we did not assign patients into a non-brace group owing to ethical reasons, because a previous study (in which our department participated) demonstrated significant differences in the changes of AVBCP between the rigid-brace and non-brace group, although this study’s protocol was slightly different than that of this previous study [13]. As our study showed a positive outcome with the rigid brace over the soft brace in the initial 12 weeks of treatment, we can also assume that the rates of vertebral collapse would be higher in patients without bracing—at least in the initial 12 weeks of treatment. However, considering that the difference disappeared after brace removal, a further large prospective study on groups with and without braces might be needed to assess the preventive effects of braces on spinal deformity. Fourth, while the rigid-brace group showed significantly higher AVBCP at 12 weeks, this difference disappeared at 24 weeks onwards. This might arise from a smaller difference in AVBCP and a larger standard deviation than we expected in our power analysis. A post hoc sample size calculation showed that to detect a statistically significant between-group difference in AVBCP of 2.5% at 48 weeks, using the standard deviations from our trial, 720 patients would be required in each treatment group. Moreover, no differences in the secondary outcomes, including improvement of back pain, QOL status (EQ-5D-3L), and JOABPEQ, were found between the groups at 48 weeks. Collectively, we consider that the between-group difference in AVBCP of 2.5%, even if statistically significant, is unlikely to be of clinical significance.

## 5. Conclusions

We found that the 12-week rigid-brace treatment for acute vertebral compression fractures did not result in statistically greater prevention of spinal deformity, better QOL, or lesser back pain than soft-brace treatment at 48 weeks. Therefore, the routine use of a custom-made rigid brace for acute vertebral compression fractures is not justified.

## Figures and Tables

**Table 1:** Baseline characteristics of the patients.

Characteristic	Rigid Brace	Soft Brace
	*n* = 141	*n* = 143
Mean age (SD), years	76.0 (5.2)	75.5 (5.4)
Mean time since fracture, weeks	1.5 (1.2)	1.5 (1.3)
No previous osteoporosis therapy, *n* (%)	21 (15)	26 (18)
No prevalent vertebral fracture, *n* (%)	107 (76)	105 (73)
Any prevalent vertebral fracture, *n* (%)	34 (24)	38 (27)
Level, *n* (%)		
T10	3 (2)	3 (2)
T11	13 (9)	11(8)
T12	45 (32)	62 (43)
L1	49 (35)	46 (32)
L2	31 (22)	21 (15)
AVBCP, (%)	72.2 (13.5)	71.4 (14.3)
EQ-5D-3L score	0.29 (0.30)	0.28 (0.28)
VAS score for back pain	72.1 (28.5)	77.4 (21.3)
JOABPEQ score		
Pain-related disorders	34.9 (30.6)	29.7 (30.6)
Lumbar function	19.8 (27.4)	18.0 (23.4)
Walking ability	21.8 (28.4)	20.9 (26.5)
Social life function	21.7 (25.6)	21.9 (26.2)
Mental health	39.1 (22.4)	38.7 (21.9)

AVBCP, anterior vertebral body compression percentage; EQ-5D-3L, European Quality of Life-5 Dimensions, 3-Level questionnaire; VAS, visual analog scale; JOABPEQ, Japanese Orthopaedic Association Back Pain Evaluation Questionnaire.

**Table 2:** Primary and secondary outcomes.

	Rigid Brace	Soft Brace	Difference (95% CI)	*p*-Value
	Mean (SD)	Mean (SD)		
**Primary outcome**				
AVBCP score				
Week 12	58.5 (15.7)	54.6 (16.3)	−3.9 (−7.8 to −0.03)	0.04002 *
Week 24	56.6 (16.9)	52.7 (16.9)	−3.9 (−8.1 to 0.4)	0.07
Week 48	55.5 (16.2)	53.0 (17.3)	−2.5 (−7.0 to 1.8)	0.20
**Secondary outcomes**				
VAS for lower back pain				
Week 12	28.9 (24.1)	29.1 (23.9)	0.2 (−5.6 to 6.1)	0.95
Week 48	28.2 (25.8)	26.3 (27.0)	−1.9 (−8.7 to 5.1)	0.43
EQ-5D-3L score				
Week 12	0.70 (0.20)	0.72 (0.18)	0.02 (−0.03 to 0.06)	0.58
Week 48	0.73 (0.20)	0.74 (0.22)	0.01 (−0.04 to 0.07)	0.67
JOABPEQ				
Pain-related disorder				
Week 12	69.5 (31.7)	73.1 (30.0)	3.6 (−3.9 to 11.1)	0.38
Week 48	57.3 (29.8)	60.6 (29.4)	3.3 (−4.4 to 11.0)	0.43
Lumbar function				
Week 12	55.9 (30.0)	60.1 (28.8)	4.2 (−3.0 to 11.3)	0.29
Week 48	66.4 (28.4)	64.8 (31.1)	−1.6 (−9.5 to 6.1)	0.91
Walking ability				
Week 12	54.7 (33.4)	55.0 (32.0)	0.3 (−7.6 to 8.3)	0.92
Week 48	61.9 (33.3)	62.3 (34.7)	0.4 (−8.5 to 9.3)	0.82
Social life function				
Week 12	50.9 (27.8)	53.6 (26.0)	2.7 (−3.8 to 9.3)	0.36
Week 48	57.7 (28.4)	63.4 (24.2)	5.7 (−1.2 to 12.6)	0.12
Mental health				
Week 12	52.1 (19.2)	54.5 (19.7)	2.4 (−2.3 to 7.1)	0.14
Week 48	55.9 (19.2)	55.6 (19.4)	−0.3 (−5.3 to 4.8)	0.96

* *p* < 0.05; AVBCP, anterior vertebral body compression percentage; EQ-5D-3L, European Quality of Life-5 Dimensions, 3-Level questionnaire; VAS, visual analog scale; JOABPEQ, Japanese Orthopaedic Association Back Pain Evaluation Questionnaire.

**Table 3:** Association of AVBCP and secondary outcome measures at 48 weeks.

Outcome	Spearman’s Rank Correlation Rho	*p*
EQ-5D-3L score	0.09	0.19
VAS score for back pain	−0.19	0.003 *
JOABPEQ score		
Pain-related disorders	0.24	0.0002 *
Lumbar function	0.11	0.37
Walking ability	0.06	0.31
Social life function	0.06	0.39
Mental health	0.01	0.87

* *p* < 0.05; AVBCP, anterior vertebral body compression percentage; EQ-5D-3L, European Quality of Life-5 Dimensions, 3-Level questionnaire; VAS, visual analog scale; JOABPEQ, Japanese Orthopaedic Back Pain Evaluation Questionnaire.

**Table 4:** Brace compliance of the rigid- and soft-brace groups within a 12-week time course.

Time	Group	4 Weeks	8 Weeks	12 Weeks
<6 h/day	Rigid brace	14	18	24
	Soft brace	7	12	22
6–12 h/day	Rigid brace	18	20	22
	Soft brace	19	22	27
>12 h/day	Rigid brace	108	97	84
	Soft brace	115	104	84
Numbers	Rigid brace	140	135	130
	Soft brace	141	138	133
*p*-value		0.30	0.49	0.77

**Table 5:** Details of the patients in both the rigid- and soft-brace groups with no medication, with non-opiate medication (aspirin, non-steroidal anti-inflammatory drugs), and with weak opiates within a 48-week time course.

Medication	Group	4 Weeks	8 Weeks	12 Weeks	24 Weeks	48 Weeks
No medication	Rigid brace	43	82	98	102	97
	Soft brace	34	77	93	94	96
Non-opiate medication	Rigid brace	86	46	29	19	17
	Soft brace	92	53	34	25	16
Weak opiates	Rigid brace	11	7	3	2	0
	Soft brace	15	8	6	5	2
Numbers	Rigid brace	140	135	130	123	114
	Soft brace	141	138	133	124	114
*p*-value		0.38	0.74	0.50	0.30	0.63
